# Future Challenges for Work-Related Health Promotion in Europe: A Data-Based Theoretical Reflection

**DOI:** 10.3390/ijerph182010996

**Published:** 2021-10-19

**Authors:** Gudrun Faller

**Affiliations:** Department of Community Health, Hochschule für Gesundheit, Gesundheitscampus 8, 44801 Bochum, Germany; gudrun.faller@hs-gesundheit.de

**Keywords:** workplace health promotion, workplace health management, digitalization, demographic change, migration, home-office, subjectification, atypical work

## Abstract

This contribution is a theoretical reflection based on statistical and empirical data as well as concepts proposed by other authors or institutions. Based on the thesis that the respective social developments equally influence and limit the orientation and design of workplace health promotion, this article deals with the challenges that arise from the contemporary social, political and economic developments for a needs-oriented and effective workplace health promotion. On the basis of a historical review of the lines of development in workplace health promotion, beginning with the Ottawa Charter in 1986, the field of tension in which work-related health promotion approaches generally operate is first outlined. Based on the results of a keyword search in relevant European statistics databases and specialized databases on the topics of demographic change, labor migration and digitalization and flexibilization of work, current development trends in the world of work are traced, priority desiderata for a future design of health promotion are derived from these, and their chances of realization are reflected upon. On the basis of the data collected, it becomes clear that today’s world of work is characterized by multidimensional diversification processes, which are accompanied by the risk of worsening social inequalities. The conclusion is that future concepts of workplace health promotion must be more universal than previous approaches, which are often limited to the focus of individual behavioral prevention. The attempt to promote workplace health promotion with economic benefit arguments also runs the risk of reinforcing social inequality. The task of effective workplace health promotion, conversely, must be to initiate critical reflection on current priorities.

## 1. Background and Conditions of Origin of Workplace Health Promotion

The Ottawa Charter [1], adopted at the first International Conference on Health Promotion in 1986, is often described as the birth of health promotion. Its main focus was initially less on the world of work than on municipal and community settings, which were among the first fields of intervention according to the setting approach within the framework of the “Healthy Cities Network” [2,3]. Nevertheless, the formulations contained in the charter are often also used as a basis for characterizing approaches to workplace health promotion.

The principles laid down in the Ottawa Charter were influenced by social movements and trends, especially the criticism of a one-sided science-oriented medicine, prevention and health education. They were supplemented by impulses and suggestions from the new social movements of the 1970s and 1980s (health, environmental, consumer, women’s, self-help movements or the civil rights movement in the United States of America) [4].

In this contemporary context, it was a novelty of the Ottawa Charter that instead of traditional health education aimed at individual behavior, structural determinants of health should be of focus, and measures should be aimed at making the social and ecological environments more health-friendly [5] (p. 118). 

Parallel to this, there have been tendencies at the level of occupational health and safety in the European Community since the 1960s, based on a broader understanding of the tasks of occupational health and safety [6]. They focused less on the prevention of damage and risks and more on the comprehensive shaping and maintenance of health in the sense of public health [7] (p. 22).

This change culminated in the adoption of the EC Framework Directive 89/391-EEC of 12 June 1989, on “the introduction of measures to encourage improvements in safety and health” and its transposition into national law in the form of the German Occupational Health and Safety Act of August 1996. At the same time and in the further course, other EU directives were transposed into national law. 

Müller [7] (p. 21) characterizes the guiding principle behind these European regulations in the sense that instead of a narrow technical orientation, authoritative supervision, a passive role for employees, and damage only in the understanding of accidents and occupational diseases, there has now been a shift toward a work environment law in which hazards are understood comprehensively, are recorded preventively, and everyone in the company can influence the design of work and its conditions.

Parallel to this change in the understanding of occupational health and safety, the first approaches to workplace health promotion had been developed in the contemporary context of the 1970s and 1980s in Germany. Promoted in particular by the research program “Humanization of Working Life”, later “Work and Technology”, new concepts were tested that were intended to contribute to the improvement of working conditions, reduce stresses, and expand employees’ scope for action [8]. Inspired by transnational influences, particularly Italian workers’ medicine [9,10] and the Scandinavian “democracy at work” model [11], participatory processes were at the heart of some of these concepts, referred to as “health circles”. In one model project, the collection of workplace health data and their joint interpretation with employees provided a basis for the participatory development of measures [12]; in the other model project, employees’ subjective interpretations of the origins of stress served as a basis for the participatory development of proposals for change [13].

## 2. Health Management as a Response to the Implementation Deficits of Health Promotion

Although the Ottawa Charter was and still is used as a normative reference point for health promotion, it was criticized in the years following its publication: its idealistic rhetoric, its low level of commitment, its failure to directly address those responsible for implementation, and the fact that it ignored all political and economic structures and power relations were criticized [14].

Rosenbrock [14] saw the resulting implementation deficits in the fact that the institutions and settings that are supposed to be oriented to the principles of the Charter all function according to criteria other than those of self-determination, participation, social justice and equal opportunities. The implementation of the principles of the Ottawa Charter should therefore be enforced contrary to traditional patterns of perception, assignment of tasks, routines of action and positions of interest, which was only partially successful.

From the 1990s onward, these strongly normative approaches to health promotion were replaced by more management-oriented concepts that demanded a higher degree of goal orientation, systematic approach and economic efficiency. The background to this development was formed by the economic and social crises in Western societies at the end of the 1970s, which resulted in a greater economization of all areas of life.

The change in objectives also manifested itself at the programmatic level of the WHO: on the basis of a comparison of the Ottawa Charter of 1986 with the Bangkok Charter [15], which is approximately twenty years more recent, Dieterich and Hahn [5] (p. 119) come to the conclusion that the project of the Ottawa Charter, which was formerly based on social criticism, has become a technocratic design project of economic thinking, in which people are reduced to consumers and social questions are only discussed as questions of management. They criticize that in the foreground of management-oriented social technologies are primarily questions of the implementation of economically validated health goals, while questions about the social distribution of power and health resources are ignored. 

At the level of workplace health promotion, there have also been increasing tendencies since the 1980s in the Federal Republic of Germany to legitimize health-related interventions by demonstrating their economic effects [16,17]. Furthermore, management principles increasingly found their way into the design of health-promoting projects and plans. Significantly in this context, Müller [18] (p. 79) describes the reorientation of the HdA (humanization of work) program in the 1980s, in the context of which the concept of innovation took over from model experiments on democratic participation and participation issues were replaced by competitive categories. 

In their conception of occupational health management, Badura et al. [19] express an understanding that sees occupational health management as an entrepreneurial task whose mission and goals consist in “lowering costs by reducing absenteeism” and in “motivating employees and binding them to the company” [19] (p. 34). Even if the presence philosophy laid down here was replaced in later publications by Badura by a positioning that sees business deficits also in “hidden” productivity losses as a result of presenteeism [20], it expresses an understanding of occupational health management that conceptualizes the promotion of employee health less as an independent value but places it primarily in the service of the business benefit calculus.

## 3. Health Promotion in the Area of Conflict between Opposing Claims

As has become clear, goals, priorities and interventions in occupational health management are not constituted independently of societal changes; they are influenced by the respective contemporary values and convictions, as well as by political and (business) economic developments and trends. The latter have direct and indirect effects on the health of employees; conversely, they shape what is considered a contemporary, normatively appropriate, effective and efficient intervention. Their inclusion in the conception of workplace health promotion represents its basis of legitimacy; at the same time, the orientation towards prevailing socio-social development directions creates specific blind spots with which science-based health and work research should deal. 

The developments in health promotion in general and workplace health promotion in particular described thus far have made clear the area of tension between normative-humanistic and economic-pragmatic orientation. While the legitimation of occupational safety and health promotion concepts in the 1970s and 1980s was based more on ethical-normative ideas, these have been increasingly replaced by management-oriented, economically driven arguments since the 1990s. Ethical–normative approaches are accused of lacking binding force and practicability. In contrast, concepts based on efficiency criteria show deficits in the form of a neglect of the target groups that should be the special focus of public health [21,22]. 

According to the definition of the Institute of Medicine [23], the task of public health is to perceive the interest of society in creating conditions in which people can live healthy lives. If it takes this task seriously, public health cannot avoid critically addressing the political-economic conditions of health and disease in the population and its subgroups, critically reflecting on its own scientific practice, and clearly positioning itself socially and politically for social justice [24].

As the critique of corresponding normative explanations makes clear, however, it is not enough to formulate postulates. From the perspective of constructively critical public health research, it is much more necessary to describe the premises under which corresponding forms of social and health inequality arise and to question the social construction conditions under which they emerge.

With this objective in mind, the following comments address selected social developments that are relevant to the field of health and work. These influences, often referred to as “megatrends,” include in particular digitalization, globalization, migration, climate change and demographic development. They are changing society and presenting it with new challenges [25]. The phenomena of flexibilization, subjectivization, and diversification that arise in the wake of these changes in the world of work raise questions about the social and health effects and the possibilities for coping with them. These questions have taken on new significance with the COVID-19 pandemic. Preliminary analyses suggest that the effects of the pandemic manifest unequally in diverse social contexts.

In the following—with special focus on the area of the European Union—the effects of selected developments related to the working environment are described in order to outline the desiderata for workplace health promotion resulting from them.

## 4. Current Changes in the World of Work

Based on the topics of “demographic development”, “migration”, “digitization” and “home office”, the following sections deal with selected challenges that current global developments pose for maintaining and promoting health in the world of work. Subsequently, postulates are formulated that can be derived from the goal of approaching a more socially just distribution of work-related health opportunities.

### 4.1. Demographic Change

Against the backdrop of falling birth rates and increased life expectancy in Europe, the proportion of older population groups is rising. This challenges the stability and performance of existing arrangements in the area of healthcare and infrastructure design [26] as well as social security systems [27]. At the same time, it is becoming more difficult to find qualified junior staff in many sectors [28]. In view of this and in view of global competition, it is a social necessity to keep aging people in the workforce [27,29].

**Figure 1:**
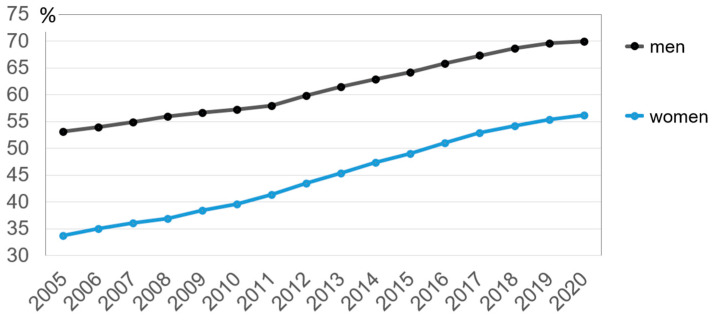
Share of employed persons in the age group 55-64 in the EU-27 (Source: [30]).

Since the end of the 1990s, increasing employment levels in older age groups and extending people’s working lives have been important objectives of national and European policy. As Figure 1 shows, the employment rate of 55- to 64-year-olds has risen steadily since 2005 at the latest and stood at 62.9% overall in 2020 [30]. However, the statistics also show that there are significant discrepancies between the genders; furthermore, these figures are still far from the targets formulated in the “Strategy Europe”, with an employment rate of 75% [31].

In this context, it is instructive to observe that the ability to work at older ages varies considerably across Europe, with the range of labor force participation in 2020 in the aforementioned age group ranging from 33.5% in Turkey to 82.4% in Sweden [30]. Furthermore, there is a clear variance depending on sectors and occupations. In particular, high physical workloads, poor work environment conditions, pronounced mental demands, and a lack of autonomy affect the ability of older people to work [27,32]. In addition, inadequate support from superiors as well as bullying or harassment by colleagues or managers seem to impair the ability to work until retirement age [33]. 

In addition to the requirements for age-appropriate design of work tasks, conditions and organization, there are also challenges in maintaining the ability and motivation to work of today’s younger employee groups. This requires medium- and longer-term concepts for aging-appropriate work design, including options for designing working lives in line with changing needs over the course of a person’s biography [34].

Possible associated conflict zones relate to questions of a perceived fair distribution of workloads between age groups, employee promotion, the understanding of leadership, the distribution of career opportunities, dealing with change, including recognition for performance, experience, and length of service [35]. 

In order to prevent disputes about these topics from becoming established as additional negative stresses and thus a factor that impairs the motivation and health of all those involved, it is advisable to expand concepts of workplace health promotion to include aspects of age- and age-appropriate life-work design as well as participation- and communication-oriented generation management.

### 4.2. Migration

In view of the challenges of the shortage of skilled workers outlined in Section 4.1, labor migration is seen as a way to compensate for the bottlenecks described. Demand exists primarily in the fields of mathematics, information technology, natural sciences and technology, as well as in nursing [36] (p. 169). 

At the European level, a fundamental distinction must be made between internal migration between EU countries and immigration from outside the EU with regard to the influx of labor. For EU citizens, the right to free movement of workers applies. With regard to the entry of third-country nationals into a Member State and their legal residence there, the conditions laid down by the EU on the basis of Articles 79 and 80 of the Treaty on the Functioning of the European Union (TFEU) apply. On this basis, Member States determine how many people from third countries may enter their territory to seek work [37]. 

Recruitment of labor from third countries in particular is described in the European Commission’s Migration Agenda [38] (p. 17) as necessary to ensure the viability of European social welfare systems in the face of demographic change, to strengthen the sustainability of social systems, and to ensure sustainable growth of the EU economy.

In the debate on labor migration, differentiation is often made with regard to the qualifications of the employees: While nationals, who would not be in competition with immigrants on the labor market due to their good education and specific expertise, low-skilled workers are attributed disadvantages because their already high unemployment risk would be increased by immigration [39]. The right-wing, which is gaining strength across Europe, also uses similar arguments and attempts to stir up fears among the population by means of constructed threat scenarios and indiscriminate mood-mongering [36,40].

However, many regions of the EU rely on the immigration of low-skilled workers, some of whom are not employed in accordance with the law (e.g., export-oriented agricultural economies in southern Spain), such that immigration, by no means, need be accompanied by increased competition for jobs [36] (p. 169).

Regardless of the qualification and the question of where the migrants have immigrated from, Baas [39] states multidimensional structural discrimination phenomena. These include the non-recognition of qualifications, the disregard of workers’ rights, disproportionate shares of atypical forms of employment, as well as precarization risks and forms of disadvantage indirectly related to work–such as access to high-quality housing or the risk of homelessness. 

In this respect, it is not surprising that according to OECD in Germany, for example, 60% of immigrant employees have left the country again after three years [41] (p. 393). Conversely, at least for some immigrants, the length of stay in the country of immigration seems to contribute significantly to an increase in the quality of work [42].

These observations make it clear that limiting oneself to recruitment strategies for labor migrants is not enough. At the company level in particular, there is great potential for retaining immigrant workers and promoting their identification with the country of immigration through appropriate integration efforts. Successful integration requires systematic integration concepts and a willingness to actively engage in the well-being of new colleagues and the development of social relationships. According to Becker [41] (p. 395) the corresponding prerequisites for success include:

- fostering an organizational culture characterized by open-mindedness and appreciation of diversity;
- offers to acquire/improve (job-related) language skills;
- the creation and implementation of an induction plan;
- practical day-to-day support;
- the use of mentors;
- the formation of tandems between immigrant and experienced employees to promote informal exchange.

As this enumeration shows, integration requires encountering the values, routines, culture of the society it is supposed to integrate and people who want to integrate it. In individualistic societies-as well as organizations-integration is made more difficult when binding generalizing standards are missing or have not been sufficiently communicated. Responsibility delegated to migrants for successful integration is more likely to be perceived by cultural aliens as an inhumane practice of leaving people alone, rather than as individualistic freedom and self-determination [41] (p. 406). 

In addition to efforts at the organizational level, it is a societal imperative and an increasingly urgent task for health promotion to address the social and ethical aspects of ever-increasing refugee movements and to develop arrangements for managing migration flows at the national and supranational levels.

### 4.3. Digitization, Flexibilization and Subjectivization

Digitization is shaping an increasing amount of areas of life and is now an intrinsic part of the world of work. The technological and social innovations associated with digitization are leading to fundamental changes in tasks, forms of work organization, activities and stresses in all sectors [43]. 

Staab and Nachtwey [44] use the term “digital Taylorism” to describe the phenomenon of transferring forms of work organization and rationalization previously typical of the industrial sector to services. With the help of close-meshed digital monitoring systems, even small errors can be systematically detected and “low performers” identified [44]. 

In addition to recording performance rates and errors, subtle monitoring technologies, for example, in the form of gamification or evaluation systems, are used to provide behavioral–psychological incentives that bring about a continuous improvement in performance [45] (p. 16).

Another phenomenon of the digital world of work is the relocation of large parts of the collaboration with colleagues, customers and clients to virtual spaces. As a result, hierarchies are dissolving, performance-based pay is gaining in importance, and entrepreneurial risks are being delegated to employees [46].

A typical example of this new form of work organization are platforms for the mediation of click-, cloud-, crowd- or gig-work. According to research conducted as part of the COLLEEM survey [45] (p. 6), around 24 million people in the EU have offered their services via platforms at least once to date. This is eleven percent of the total working EU population. For three million Europeans, this is the main source of income.

The example of this fast-growing field of employment shows that employees are increasingly becoming entrepreneurs of their own labor. Entrepreneurial thinking is considered a key competence of tomorrow’s working world [46,47]. 

Closely related to the increasing flexibilization and subjectivization of work is the spread of so-called “atypical employment relationships”, which are mostly understood to mean forms of employment that do not conform to the standard or “typical” model of full-time, regular, open-ended employment with a single employer over a long time span. Atypical work includes part-time work, temporary work, fixed-term work, casual and seasonal work, self-employed people, independent workers and homeworkers [48]. There is agreement with regard to the statement that some types of atypical work have a higher risk than others, and the degree of risk is influenced by the situation of the individual [48,49].

According to Schulze-Buschoff [50], 36.4% of all employed persons in the EU28 were already working in atypical constellations in 2014, with considerable differences between the individual countries. 

Although it is repeatedly emphasized that atypical jobs are not to be equated across the board with precarious employment relationships, it is evident that the incomes of atypical employees are generally below average, and their career biographies are characterized by considerable discontinuities as well as an increased risk of unemployment [50]. 

More often than regular jobs, atypical forms of employment are characterized by unfavorable working conditions (monotony, dangerous activities, limited room for maneuver, lack of involvement in the company) [49]. 

In countries where trade union membership is linked to affiliation with a company, there are often hardly any opportunities for representation of interests in certain forms of atypical employment. In systems where social security is insurance-based and thus oriented toward the equivalence principle (e.g., Germany, Italy), atypical employment is also associated with the risk of old-age poverty [50].

Country-specific surveys within the EU show that COVID-19 exacerbates the problems associated with precarious work and increases the vulnerability of temporary workers, the self-employed and those working under “hybrid” arrangements. The worsening of social disparities has been observed at both regional and national levels [51]. 

Recommendations to date for improving the protection of employees in atypical or precarious employment relate to better social security for those affected, for example in the form of compulsory insurance for old age and unemployment and in the form of affordable health insurance contributions in the case of solo self-employment, and also to the replacement of mini-jobs with part-time contracts that are subject to tax and contributions [52]. In the case of temporary work, there are also calls for increased welfare obligations, on the part of both the hiring and the borrowing companies, relating to occupational health and safety, company integration, communication, qualification and representation of interests, among other things [53]. Beyond the company level, counseling and support services as well as new forms of collective interest representation could improve the situation for those affected by precarious work [53,54].

### 4.4. Work in the “Home Office“

The spread of mobile forms of work was massively accelerated by the corona pandemic. This is particularly true for “home office” work. While only 5.4% of employees in the EU-27 usually worked from home in 2019, Eurofound estimates this share to be 40% in 2020 [55].

With the possibilities of a more flexible organization of work and time and the inherent tendencies towards result-oriented control, virtual forms of work organization promise more self-determination for employees, while at the same time, they agree with new performance controls, work intensification and an overlapping of private life worlds by work-related demands [56]. 

In addition to these now more frequently discussed consequences of flexibilization, the debate about the “home office” often neglects the characteristic of its social selectivity: During the COVID-19 pandemic, for example, by no means all employees were able to protect themselves from infection by relocating their work. Intellectually active, highly skilled, and high-earning workers generally had easier access to the “home office”, while low-skilled, physically working people faced not only the risk of infection but, in addition, the risk of lost income due to short-time work or unemployment [56,57].

In addition, the pandemic has exacerbated existing gender inequalities: for female employees, the closure of daycare centers and schools despite “home office” has been accompanied by a huge increase in the double burden of paid work and care work [56,58]. 

**Figure 2:**
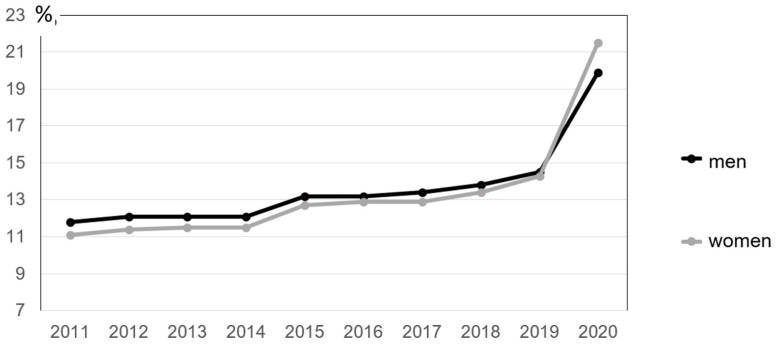
Shares of employed men and women working from home (occastionally and usually). Source: [59], own calculations.

Figure 2 shows that the share of employees in the EU-27 who occasionally or usually work from home has risen steadily for both genders, with the percentage of women exceeding that of men for the first time in 2020.

Impulses for improving the situation of mobile or “home office” employees relate to the inclusion of mobile work arrangements in relevant occupational health and safety regulations, the prohibition of performance and behavioral controls through collective agreements, the demand for working time recording to prevent the dissolution of boundaries, and recommendations in favor of maintaining social relationships in the company. However, these proposals do not cover more far-reaching issues, such as how newly emerging forms of inequality, for example, with regard to access to “home office” or in the form of the double burden of care work and gainful employment and thus gender-specific stress discrepancies, can be avoided or compensated for.

## 5. Conclusions

The trends highlighted thus far, such as demographic development, immigration movements, digital innovations and their fundamental effects on the design of forms of work, places of work and working hours, right up to the shift in the attribution of responsibility and the intensification of social inequality, make it clear by way of example that future concepts of workplace health promotion will have to be thought of more universally than previous concepts, which have often been limited to a focus on health in the sense of adherence to individual behavioral prevention, have provided. 

One approach that is currently being strongly promoted in the context of digitalization is the attempt to provide employees with apps that optimize their behavior and state of mind and encourage them to use corresponding programs to ensure their individual work and performance capacity by correcting their own health behavior. The problem with such approaches is not only the fact that the responsibility for health at work is assigned unilaterally to the employees and the question of the need for corrections on the part of the working conditions remains untouched [60]. Corresponding individual preventive behavioral interventions also neglect the opportunity to generate impulses in favor of age-appropriate and diversity-friendly organizational development options and to use these in the sense of a self-reflexive change and improvement dynamic at the company level. Furthermore, questions of social inequality in the context of diversifying forms of work organization and design as well as questions about the distribution of power remain unresolved in these approaches.

One of the requirements to be placed on future workplace health promotion is that the interests of a workforce characterized by diversity must be included—this, under the premise that disadvantages power imbalances must be reduced.

Diversity-oriented workplace health promotion in the sense of interest-driven discursive organizational development also means that the effects of forms of work organization characterized by new technologies are recorded in the sense of the assessment of working conditions required by the European Framework Directive on Occupational Health and Safety (Directive 89/391/EEC) and that suitable measures are implemented with the participation of those affected to reduce the negative consequences of stress and to ensure that work is designed in a humane manner. 

In order to ensure the binding nature, professionalism and conditions for success of correspondingly expanded occupational safety and health promotion concepts, it is important to strengthen the presence and influence of workplace interest groups as well as occupational safety and health promotion experts, to clarify their roles and to promote their cooperation.

Health promotion at the company level must be supplemented by supra-company, work-related support structures that are designed to strengthening the social security, representation of interests and health protection of contractors who are not integrated into company organizations, in view of the blurring boundaries between work and reproduction, to consider the sphere of private care work–which is currently completely excluded from the debate–and to develop suitable concepts for this which include relief options and conversely enable a fairer distribution of paid and unpaid work between the sexes.

In view of the current challenges described in the preceding sections, the question arises as to the chances of realizing an opportunity- and health-oriented design of work. These are characterized by the tension between ethical orientation and economic benefit considerations, as discussed at the beginning of this article.

The argumentation discussed here and still used in some of the specialist literature, that safety and health at work are promoted by economic benefit calculations on the part of companies, cannot resolve this conflict due to its selective orientation; rather, corresponding approaches favor the already more privileged part of the workforce, while originally already disadvantaged groups are usually not covered by corresponding strategic economic considerations, such that social and health inequality tends to increase against the background of economically narrowed concepts.

Attempts to implement elements of social protection and solidarity via minimum standards for remuneration and working hours, compulsory social insurance and other protective rights are still in their infancy at the moment. Initiatives from the political arena to strengthen the rights of employees in precarious or atypical employment are in place, although the prospects of success are currently considered uncertain [56].

It remains to be seen whether it is possible to establish the necessary conditions for a fair distribution of work-related health requirements for all employees within a system of values and economics that is based on dynamic growth, global competition and the exploitation of natural resources. The task of work-related health promotion should therefore be to initiate a critical reflection on these priorities in order to enable new accentuations in the sense of a humane, just and sustainable design of working and living conditions.

## Data Availability

Publicly available datasets were analyzed in this study. This data can be found here: https://appsso.eurostat.ec.europa.eu/nui/show.do?dataset=lfsa_ehomp (accessed on 20 September 2021) and https://ec.europa.eu/eurostat/databrowser/view/LFSI_EMP_A__custom_1116266/default/table?lang=de (accessed on 20 September 2021).
